# The Situation of Safe Surgery and Anaesthesia in Tanzania: A Systematic Review

**DOI:** 10.1007/s00268-018-4767-7

**Published:** 2018-08-20

**Authors:** Karolina Nyberger, Desmond T. Jumbam, James Dahm, Sarah Maongezi, Ahmed Makuwani, Ntuli A. Kapologwe, Boniface Nguhuni, Swagoto Mukhopadhay, Katherine R. Iverson, Erastus Maina, Steve Kisakye, Patrick Mwai, Augustino Hellar, David Barash, Cheri Reynolds, John G. Meara, Isabelle Citron

**Affiliations:** 1 Program in Global Surgery and Social Change, Department of Global Health and Social Medicine, Harvard Medical School, Boston, MA USA; 2 Ministry of Health, Community Development, Gender, Elderly and Children, Dodoma, United Republic of Tanzania; 3 President’s Office, Regional Administration and Local Government, Dodoma, United Republic of Tanzania; 4 Dalberg Advisors, New York, NY USA; 5 The G4 Alliance, Chicago, IL USA; 6 Jhpiego, Baltimore, USA; 7 GE Foundation, Boston, MA USA; 8 Assist International, Ripon, CA USA; 9 Boston Children’s Hospital, Boston, MA USA; 10 WHO Collaborating Centre for Surgery and Public Health, Department of Clinical Sciences in Lund, Lund University, Lund, Sweden

## Abstract

**Background:**

Improvement in the surgical system requires intersectoral coordination. To achieve this, the development of National Surgical, Obstetric, and Anaesthesia Plans (NSOAPS) has been recommended. One of the first steps of NSOAP development is situational analysis. On the ground situational analyses can be resource intensive and often duplicative. In 2016, the Ministry of Health of Tanzania issued a directive for the creation of an NSOAP. This systematic review aimed to assess if a comprehensive situational analysis could be achieved with existing data. These data would be used for evidence-based priority setting for NSOAP development and streamline any additional data collection needed.

**Methods:**

A systematic literature review of scientific literature, grey literature, and policy documents was performed as per PRISMA. Extraction was performed for all articles relating to the five NSOAPS domains: infrastructure, service delivery, workforce, information management, and financing.

**Results:**

1819 unique articles were generated. Full-text screening produced 135 eligible articles; 46 were relevant to surgical infrastructure, 53 to workforce, 81 to service delivery, 11 to finance, and 15 to information management. Rich qualitative and quantitative data were available for each domain.

**Conclusions:**

Despite little systematic data collection around SOA, a thorough literature review provides significant evidence which often have a broader scope, longer timeline and better coverage than can be achieved through snapshot-stratified samples of directed on the ground assessments. Evidence from the review was used during stakeholder discussion to directly inform the NSOAP priorities in Tanzania.

## Background

**Fig. 1:**
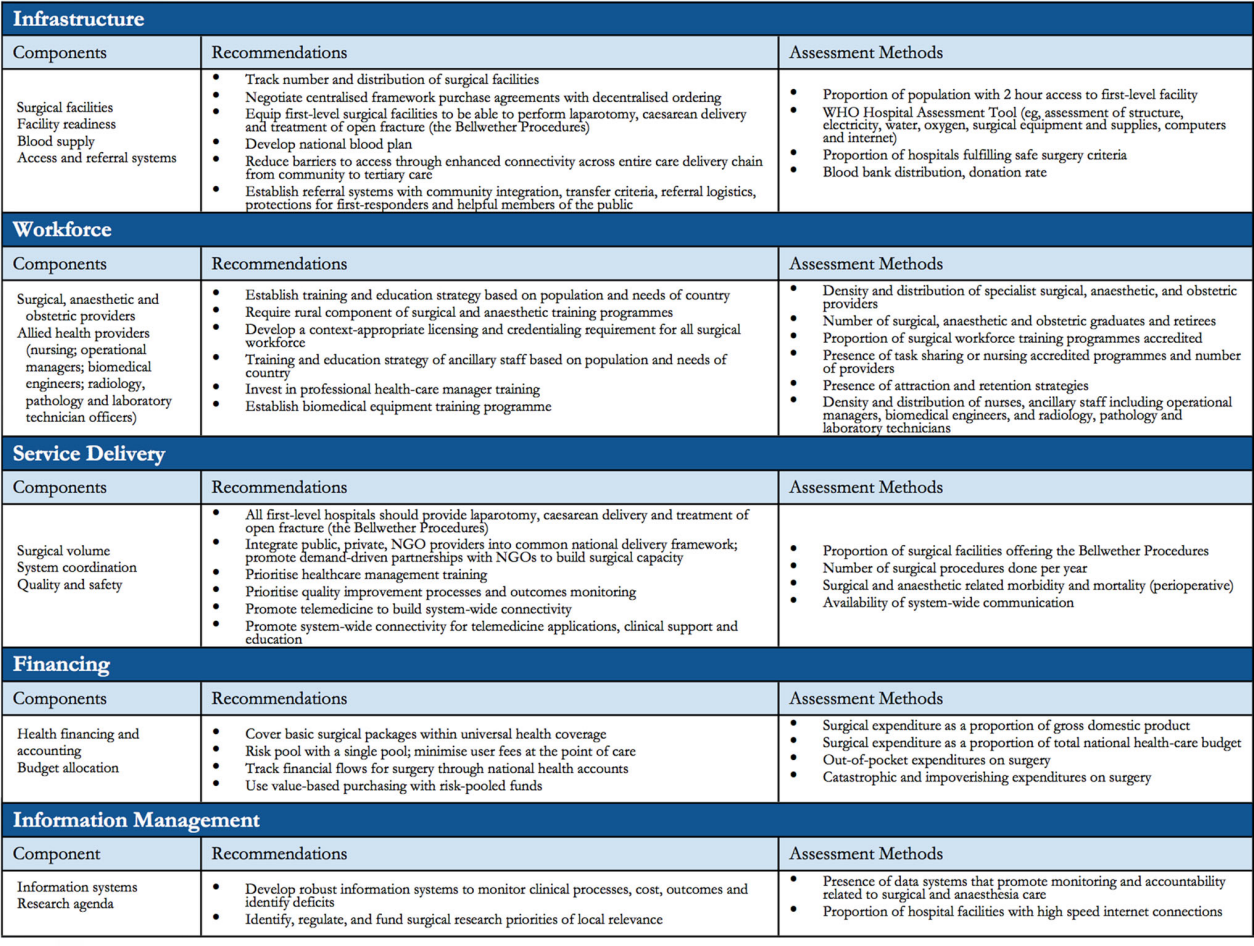
The national surgical, obstetric, and anaesthesia plan template proposed by the Lancet Commission on Global Surgery [4]

Surgery has become a global health priority with the adoption of the World Health Assembly Resolution 68.15 that called for the strengthening of emergency and essential surgical and anaesthesia care as an integral component of Universal Health Coverage (UHC) [1]. Additionally, the publication of the Lancet Commission on Global Surgery (LCoGS) report in 2015 has proved to be a springboard for global surgery advocacy. The report highlighted the fact that 5 billion people lack access to safe, timely and affordable surgical and anaesthesia care and 143 million additional surgical procedures are needed each year to attend to the unmet need [2]. In recognition of the fact that improving surgical care requires intersectoral (between ministries), cross-cutting (across multiple ministry of health departments) healthcare coordination, LCoGS recommended the development of National Surgical, Obstetric, and Anaesthesia Plans (NSOAPs) to serve as a roadmap to improvement across five domains: service delivery, infrastructure, workforce, financing, and information management (Fig. 1) [2, 3]. LCoGS developed a framework of strategic areas to be addressed within each of these domains.

In late 2016, the Permanent Secretary of the Ministry of Health, Community Development, Gender, Elderly and Children (MOHCDGEC) in Tanzania issued a directive for the creation of a NSOAP. One of the first steps of strategic planning in the health sector is conducting a situation analysis to identify the strengths and weakness of the health system to inform priority areas [4].

The aim of the study was to review the current situation of the five domains of surgical systems strengthening in Tanzania to inform the development of each domain of the Tanzanian NSOAP [2, 5]. This study represents the first comprehensive review of a national surgical system using the LCoGS NSOAP framework through a systematic review of scientific and grey literature. As such, one of the goals of this review was to show how systematic literature reviews can be used to inform process and priority setting in the development of a NSOAP.

## Methods

### Study Design

**Fig. 2:**
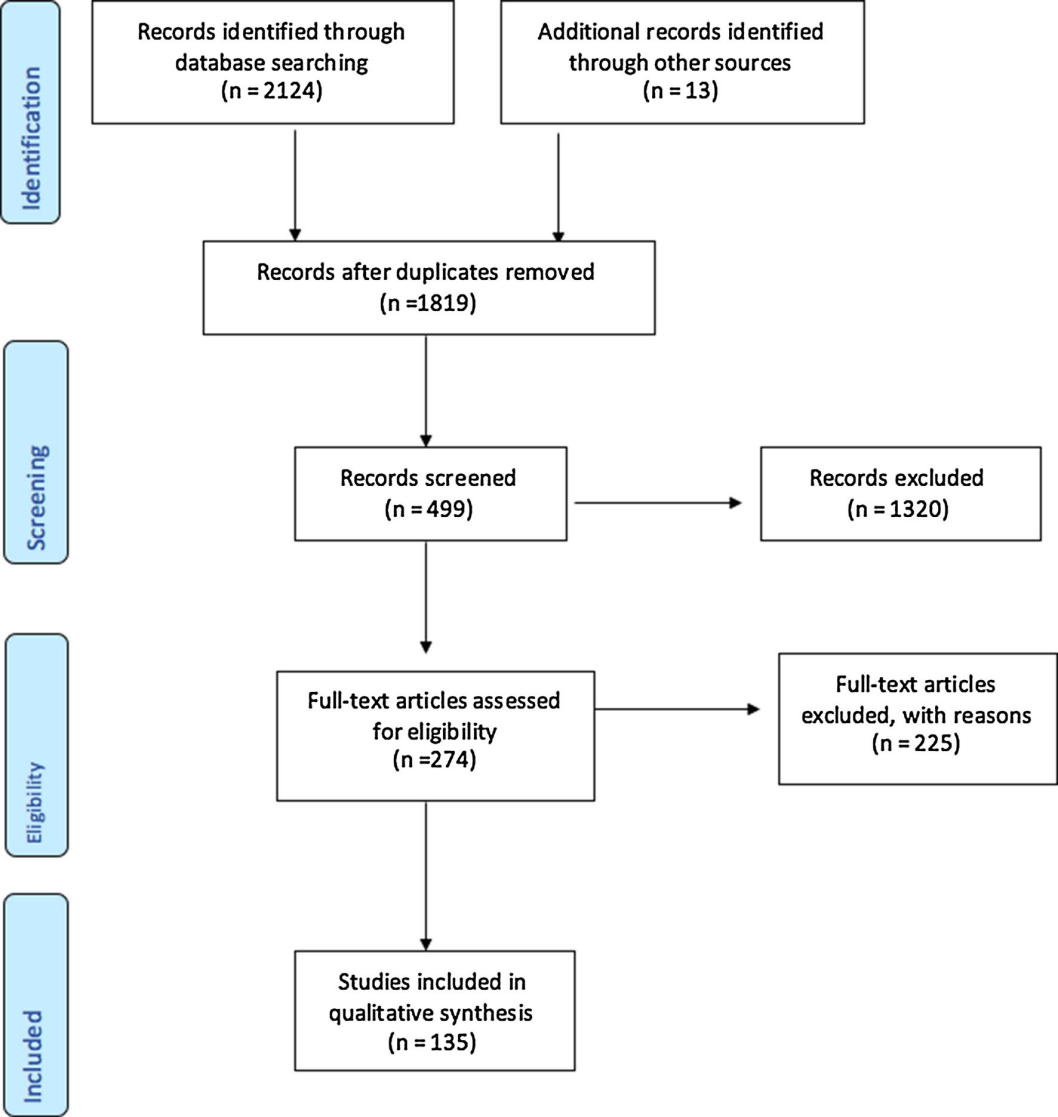
PRISMA flow diagram

A systematic literature review was performed on published scientific literature, grey literature, policies and policy guidelines in Tanzania. The review was conducted between November 2016 and March 2017 according to the PRISMA guidelines (Fig. 2) [6].

### Search Strategy and Screening

The search aimed to capture all publications relating to Tanzania and the five LCoGS surgical domains. The key themes reviewed are presented in Fig. 1. A full list of search terms used is included in “Appendix”. Searches were made in PubMed, Embase, and African Index Medicus. Policies, and policy guidelines, health data from MOHCDGEC, civil society organization reports, and parliamentary speeches were also reviewed [7]. Personal contacts were made to seek additional documents and data points. Google was used to search for grey literature.

### Data Collection and Screening for Eligibility

Following an initial search, titles were screened for duplicates by three authors (IC, JD, DJ). The resulting abstracts were then screened for eligibility by four authors (IC, JD, DJ, KN). Eligible articles were abstracted by all authors using a custom abstraction tool.

### Eligibility Criteria

Included in this study were all articles in the English language published from January 2003 to January 2017 (Fig. 2). Case reports were excluded from the review.

### Primary Outcome

The outcome of interest was any quantitative and qualitative data relating to the five domains in the NSOAP framework.

### Analysis

The abstracted data were summarized into five policy briefs designed to efficiently brief NSOAP stakeholders about the state of the surgical healthcare system in Tanzania to allow for evidence-based discussion during the NSOAP priority setting workshops. In this review, findings are presented in accordance with the LCoGS national surgical planning framework.

## Results

Our search generated 1819 unique articles. Title and abstract screening yielded 274 articles. Full-text screening produced 135 articles, of which 46 were relevant to surgical infrastructure, 53 to workforce, 81 to service delivery, 11 to finance, and 15 to information management. Some of these articles were relevant to multiple domains. Of the 135 relevant articles; 76% originated from Tanzanian institutions, 48% had a Tanzanian first author, and 81% had at least one author from Tanzania. A summary of the findings can be found in Table 1.

**Table 1:** Summary of NSOAP recommendations derived from the systematic review

Domain	Finding	Recommendation
Equipment and consumables	20% of dispensaries, 25% of health centres and 45% of hospitals had minimum surgical equipment for their level. 68% of hospitals had necessary medicines and commodities Half of the facilities performing major surgery have blood transfusion, ultrasound, and X-ray capabilities	Define and procure appropriate equipment and consumables at each level of care
Blood supply	71.8–82.9% of blood ordered is unused	Develop and implement guidelines around blood prescribing and usage National blood plan to increase donation and distribution
Education	0.31 physician surgeons, obstetricians, and anesthesiologists per 100,000 population of the recommended 20–40 per 100,000 population 39.6% of tracked medical graduates not practicing clinical medicine 41% of practicing doctors located in urban regions	Minimum staffing guidelines to include surgical, anaesthesia and obstetric clinicians. Increase access to training programs Increased sponsorships for internships and residencies.
Task-shifting practice	Non-obstetric major surgical procedures in Tanzania performed by: 55.8% non-physicianclinicians (NPCs) 28.7% surgical specialists 15.5% medical officers [17]	Define and regulate the role of non-specialist providers Develop supportive supervision networks of non-specialist staff
Referral system	70% of over 11,000 patients seen at Muhimbili National Hospital self-referrals	Each region to develop a referral plan including transfer criteria, referral logistics, and community education and outreach.
Budget allocation	5.6% of Tanzania’s GDP spent on health in 2014 Budget allocation to surgical care is unknown	Advocate for 15% of GDP health spend as per the Abuja declaration. Track % of health budget spent on surgery Trackcosts for providing surgical care
Patient expenditure	27% of health spending was from out-of-pocket expenditure 65.8% of population at risk of catastrophic expenditure from seeking surgical care	Monitor cost of surgical care to the individual patient. Advocate for inclusion of essential surgical anaesthesia and obstetric care as part of national health insurance schemes
Data collection	National Health Management Information System (HMIS) collects data on a limited number of surgical and anaesthesia indicators, including available workforce by district and region and surgical procedures like caesarean sections	Integrate the NSOAP monitoring and evaluation framework in HMIS to ensure visibility of surgical indicators on the national dashboard

### Service Delivery

Eighty-one studies explored surgical service delivery in Tanzania. Nevertheless, no studies were found that systematically assessed the national capacity to delivery of safe surgery, obstetrics, and anaesthesia (SOA) care. According to the World Bank’s World Development Indicators, Tanzania performs 484 surgical procedures per 100,000 population per year [8]. In 2003, only 35% and 23% of obstetric needs were met in Mwanza and Kigoma regions, respectively [9]. Similarly, according to Galukande et al., the unmet need of caesarean section in Bagamoyo district in Pwani region was 65% [10]. In a study conducted in Tanzania, Uganda and Mozambique, non-obstetrical surgical procedures accounted for 40–60% of surgical procedures at the district level, of which 60% were major surgical procedures [10]. Furthermore, the 2012 Service Availability and Readiness Assessment (SARA) report noted that 21% of dispensaries, 47% of health centres, and 79% of hospitals in Tanzania provided basic surgical care [11]. Of these, only 27% of dispensaries, 33% of health centres, and 51% of hospitals were deemed to be “ready” for this service, as defined by having correct personnel and equipment to provide a safe service.

Several reports noted inefficiencies in the current referral system [12, 13]. For example, in 2004, 70% of patients seen at Muhimbili National Hospital were self-referred, 67% of which presented with surgical conditions, and 96% stated the reason for self-referral as lack of expertize at the district hospital [12]. Moreover, the Primary Health Services Development Program (PHSDP) 2007–2017 published by the MOHCDGEC classified the referral system as “non-functional” due to system limitations that led to inappropriate referrals [13].

### Workforce

Fifty-three studies explored the SOA workforce in Tanzania. Most studies regarding SOA workforce noted workforce shortages at all levels of care [9, 14–16]. At the time of the review, the specialist surgical workforce density of Tanzania was 0.31 per 100,000 population [2, 8]. A majority of SOA care is provided by non-physician clinicians (NPCs) [9, 17]. In Kigoma and Mwanza regions, excluding the university hospital, 85% of caesarean sections and other obstetric surgeries are performed by NPCs [9]. In 2012, 55.8% of non-obstetric major surgical procedures in Tanzania were performed by NPCs, followed by surgical specialists (28.7%) and medical officers (15.5%) [17].

At the time of this review, there were only 22 anaesthesiologists, equivalent of 0.05 specialist anaesthesiologists per 100,000 population [8, 15, 18]. Like surgical and obstetric care, most anaesthesia care is administered by NPCs [16, 19]. In 2012, 87% of anaesthesia providers in the country were NPCs [16]. Adding NPC anaesthesia providers brings the anaesthesia provider density to 0.15 per 100,000 population [19].

A 2012 census found that 41% of practicing doctors in Tanzania were located in major urban cities, where medical training and research institutions are located [20]. Penoyar et al. [16] also found that 88% of surgical specialists identified in their national survey were employed by the six largest hospitals in the country.

One modelling exercise by Goodell et al. [21] found that given the current admission rates and no attrition, Tanzania would have about 2.6 practicing doctors per 10,000 population by 2025 compared to the 260 per 100,000 recommended by the WHO, although we note that this metric has since been updated by the WHO to include all skilled healthcare staff [22]. However, they predicted that only 44% of Tanzanian-trained doctors will be working in clinical practice by 2025 primarily due to doctors taking up non-clinical positions.

### Infrastructure

**Fig. 3:**
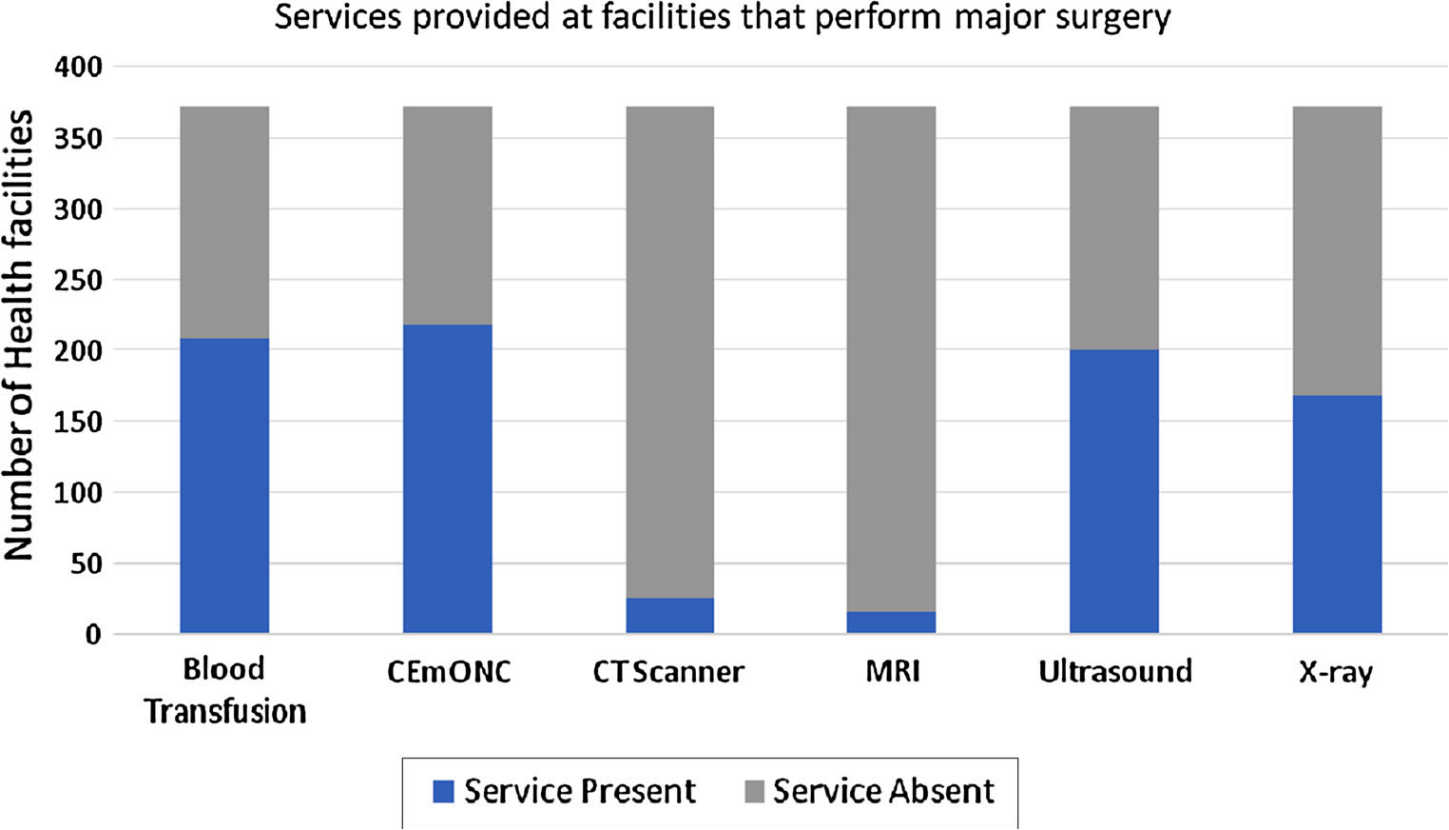
Availability of services at health facilities that provide major surgery. Data obtained by MOHCDGEC’s Health Facilities Registry (HFR)

Forty-six studies explored the surgical infrastructure in Tanzania. In a nationwide assessment of 48 facilities providing surgical services and serving 46% of the population, Penoyar et al. [23] found that only 42% had consistent access to oxygen and 37.5% had both consistent supply of running water and electricity. Similarly, the 2012 SARA report showed that of facilities providing basic surgery, only 20% of dispensaries, 25% of health centres and 45% of hospitals had the minimum appropriate surgical equipment, and only 68% of hospitals had appropriate medicines and commodities [11]. Data obtained from MOHCDGEC’s Health Facilities Registry showed that only half of the facilities performing major surgery have blood transfusion, ultrasound, and x-ray capabilities (Fig. 3) [24].

Access to safe blood and blood products at health facilities was identified as a challenge by several studies [18, 25–27]. In 2016, only 36% of all blood need in the country was met [25]. Wastage of blood and blood products was also noted by some studies. Chalya et al. [26] at Bugando Medical Centre, a zonal hospital in Mwanza region, found that up to three-quarters of cross-matched blood at the hospital was unutilized. Similar blood utilization ratios were reported at Muhimbili National Hospital [27].

### Information Management

Fifteen studies explored the information management of surgical care in Tanzania. Research studies on information systems specific to SOA were scant. An audit conducted in Kilimanjaro Christian Medical Centre found that patient records were often only partially completed with sections like procedures and follow-up completed 27% and 0% of the time, respectively [28]. A review of government policies and databases revealed that a limited number of surgical data are currently being collected at the national level [24]. None of the six LCoGS indicators are routinely collected and reported at the national and international levels [8].

### Financing

Eleven studies explored the financing of surgical care in Tanzania. In fiscal year 2014, the total expenditure on health per capita for Tanzania was US$ 52 (current US$) accounting for 5.6% of GDP, below the 2001 Abuja declaration target of countries allocating 15% of annual budget to health [8, 29]. The proportion of healthcare expenditure attributed to surgical care was not found in this review. However, a survey of eight hospitals in Mozambique, Tanzania, and Uganda found that only 7–14% of operational costs at these facilities was allocated to surgery [30]. A majority of surgical operational costs were attributed to obstetric surgery with personnel (60.3%) being the largest cost category [30]. By conservative estimates, the study found expenditure on surgery to range from US$0.06 to US$0.19 per capita.

The current proportion of the population at risk of catastrophic (defined as greater than 40% of annual household income) and impoverishing (defined as forcing a family below the poverty line) expenditures from seeking surgical care is estimated to be 65.8% and 85.5%, respectively [8].

## Discussion

The data found in this review highlight priority policy areas for improving the Tanzanian surgical system. The most important finding of the review is that despite little systematic data collection around SOA care specifically, a thorough literature review can provide significant evidence from a broad range of sources. In Tanzania, the wealth of existing evidence, particularly from existing capacity assessments such as the SARA, led to the NSOAP core committee to cancel a previously scheduled baseline capacity assessment. This had four key advantages: (1) the approach saved time and kept momentum in the NSOAP process, (2) it saved considerable resources of both transport time and facility providers time, (3) it avoided duplication and leveraged existing efforts, and (4) the range of data sources was broader than would have been possible in a prospective assessment of hospital capacity. Peer-reviewed and long-term outcomes studies added depth to what is often a snapshot assessment of capacity. The data granularity proved more than adequate to inform priority setting in that high levels of detail, for example the exact hospitals where an anaesthesia machine is needed, is needed later during implementation but should not be a barrier to priority setting when so many obvious needs exist.

The World Health Organization (WHO) recommends that priority setting in the development of national strategic plans be as far as possible evidence-based [31]. The data provided in this review, supplemented by key informant interviews, focus groups, and workshops not presented in this paper, were summarized into policy briefs which were used by policy makers during the NSOAP development process. These policy briefs allowed for evidence-informed priority setting during the NSOAP development process. The following sections detail how the results from this systematic review were used to inform priority setting in the Tanzanian NSOAP and are summarized in Table 1. Although additional informal interviews were performed by the NSOAP development team, this paper focuses only on information obtained from the systematic review.

### Service Delivery

Upgraded health centres provide major obstetric procedures like caesarean sections while district hospitals provide more complex essential and emergency surgeries (Table 2) [24]. Comprehensive emergency and elective surgeries are performed at regional, zonal, and national hospitals [32, 33]. This review identified severe limitations in surgical service delivery at all levels of care, and as such, the NSOAP aimed to strategically improve capacity for surgical care provision from the community level to the zonal and national levels. The NSOAP recommends that all first-level hospitals provide the Bellwether procedures (exploratory laparotomy, open fracture treatment, and caesarean section). Inefficiencies in the SOA referral system were found to be primarily due to workforce and infrastructure constraints at lower-level facilities [13]. To address these inefficiencies and improve the SOA referral system, the Tanzanian NSOAP seeks to provide consistent high-quality SOA services for basic emergency care at lower-level facilities. Consistent service provision will likely strengthen the referral system by improving patient distribution and reducing self-referrals. Moreover, the NSOAP aims to further strengthen the referral pathway by standardizing referral guidelines and improving patient transportation such as training personnel on the use of ambulances and transportation of patients [34–36].

**Table 2:** Number of facilities by level providing major surgical care in Tanzania as reported in the National Health Facility Registry

Hospital level	Number providing major surgery	Total	Percentage (%)
Zonal, national, or specialized	6	7	86
Regional hospitals	18	22	82
District hospitals	85	85	100
Health centre	104	586	18
Dispensary	72	4249	2
Other or unspecified	82	95	86

Data provided by the MOHCDGEC

### Infrastructure

In Tanzania, the availability of surgical infrastructure, equipment, and supplies varies by health facility level and geographic location but is generally inadequate [11, 18, 19, 24, 37]. Essential basic infrastructure like clean water, electricity, and oxygen is often unreliable at many health facilities providing surgical services. Thus, the final NSOAP details minimum surgical and anaesthetic equipment and consumable for each facility level. Furthermore, the NSOAP lays out a clear strategy for procuring and upgrading all health facilities providing SOA services with necessary infrastructure and equipment.

In addition to low donation rates, the systematic review identified significant blood wastage as a challenge [26, 27]. Therefore, strategies to increase blood availability through improved usage, by developing and disseminating blood utilization guidelines, are also detailed in the final NSOAP.

### Workforce

**Fig. 4:**
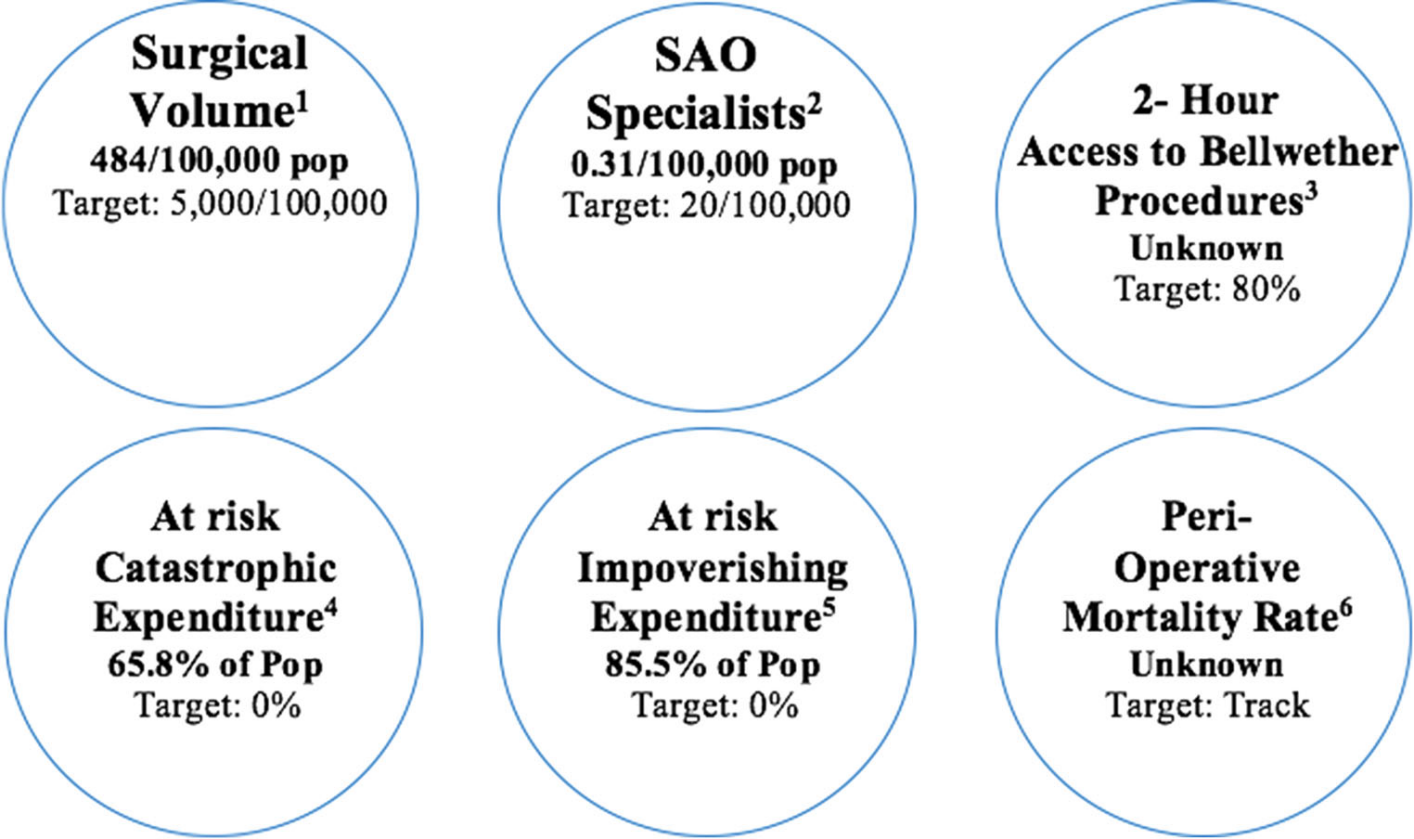
Six Core Lancet indicators for monitoring access to safe, affordable surgical, and anaesthesia care when needed in Tanzania. ^1^Procedures done in an operating theatre, per 100,000 population per year; ^2^number of specialists surgical, anaesthesia, obstetric physicians who are working, per 100,000 population; ^3^proportion of the population that can access, within 2 h, a facility that can do the Bellwether procedures; ^4^proportion of households protected against catastrophic expenditure from direct out-of-pocket payments for surgical and anaesthesia care; ^5^proportion of households protected against impoverishment from direct out-of-pocket payments for surgical and anaesthesia care; ^6^all-cause death rate prior to discharge among patients who have undergone a procedure in an operating theatre, divided by the total number of procedures

A concerning shortage of SOA workforce in Tanzania was noted in this review, with only 0.31 specialist surgical workforce per 100,000 population (Fig. 4) [8]. This shortage is further compounded by an inequitable distribution countrywide [20]. To address these workforce challenges, first, the NSOAP aims to increase training capacity for specialist surgery, obstetric, and most critically anaesthesia providers by strengthening current training institutions and developing new training programs. Secondly, minimum staffing guidelines for health facilities in Tanzania are to be updated to include the surgical workforce necessary for each level of care such that posts can be created for uptake of all trained specialists. Thirdly, the NSOAP addresses retention schemes to ensure surgical providers remain in clinical practice [21]. Some strategies for rural retention have been recommended by the WHO and were adapted to the Tanzanian context in the final NSOAP. For example, the NSOAP recommends the deployment of SOA workers as functional clusters to ensure surgeons and obstetricians are deployed with anaesthesia staff to ensure they can work safely, and in a minimum of pairs to avoid burnout and allow for consistent service delivery for patients, a key factor in referral pathway strengthening. The NSOAP makes financial provisions for rural wage supplements for SOA specialists and allied health professionals, as well as a curriculum for supportive supervision to ensure that rural workers have access to continuing medical education, a factor known to be most strongly predictive of rural retention [38]. It is hoped that by taking a health systems strengthening approach, improved conditions through access to support from a functioning regional SOA hub, improved supply chain, improved staffing and infrastructure will also make conditions for the rural workforce more sustainable.

With the severe shortage of specialist SOA providers, NPCs play an important role in delivering surgical care in Tanzania. Therefore, in developing the NSOAP, the MOHCDGEC identified strategies for supporting safe practice and regulation of NPCs such as regular supportive supervision and continuing medical education opportunities for NPCs [39]. Additionally, the NSOAP aims to increase the number of NPCs in anaesthesia through curriculum standardization, expansion of training, and employment opportunities.

### Information Management

This review found information management for SOA care to be severely lacking. One strategy adopted in the NSOAP to improve data collection and reporting at the facility level is to introduce standardized operating theatre and post-operation ward registers to all health facilities along with data collection training sessions. The NSOAP equally aims to collaborate with the National e-health strategy and Health Management Information System (HMIS) to ensure that surgical information is incorporated into current electronic data capturing systems such as the District Health Information Systems 2 (DHIS2) [40]. In addition, interventions on prospective collection and reporting of the six global surgery indicators recommended by the LCoGS and adopted by the World Bank and WHO are included in the NSOAP [2].

### Financing

Two broad areas of focus for improving financing of surgical systems in Tanzania include increasing financial resource allocation to SOA care and implementing strategies to reduce catastrophic and impoverishing expenditure to patients. Accordingly, the NSOAP finance section prioritizes the tracking of national budgetary allocations to surgical services including systemic and direct costs for providing surgical care as part of the national health account.

To address the issue of widespread financial risk demonstrated by the WDI indicators, the NSOAP advocates for a comprehensive inclusion of SOA procedures in national health insurance schemes such as the National Health Insurance Fund (NHIF) and the Community Health Fund (CHF). Such strategies would protect patients by reducing out-of-pocket (OOP) expenditure by patients receiving SOA care and equally ensure the health facilities are appropriately reimbursed for the surgical services they provide.

### Limitations

Although this systematic review captured a comprehensive amount of information on the surgical system in Tanzania, the authors acknowledge that many other policy areas, not captured by this systematic review, are required for a NSOAP to be comprehensive and to strengthen the entire surgical ecosystem. Therefore, additional research may be required prior to the development of the NSOAP to fill in gaps highlighted by the systematic review, as well as more in-depth research after the NSOAP drafting to guide specific program implementation. In our experience, the particular areas of where data were still required during the NSOAP drafting were representative figures around current workforce and training capacity. However, this was supplemented by the working knowledge of the expert working group. Although the study assessed scientific and grey literature, there is a significant risk of publication bias with the information making it to publication more likely to reflect academic tertiary centres or areas with links to NGOs who were often the authors of these reports. The literature review contained information from the last 15 years and therefore some of the information may be outdated. It must also be accepted that not every recommendation of the NSOAP will be based in scientific evidence and some may be formed by the consensus view of the cross-sectional stakeholders involved in the NSOAP process.

This systematic review is based on the LCoGS framework and therefore reflects the priorities ascertained at that time by the consensus of the LCoGS expert working group. This framework is unlikely to be a perfect “one-size-fits-all” template and therefore should be adapted for each context as well as routinely reviewed as real experience and lessons learnt around NSOAP development emerge. For example, although the LCoGS template has just five domains, the NSOAP in Ethiopia was expanded to eight pillars and the final NSOAP in Tanzania added a sixth pillar for governance [3, 41]. In the future, it is likely that the template is iterated, for example to contain a stronger emphasis on community engagement and health-seeking behaviours, which is currently only a subsection of the service delivery domain but may justify more prominence. To ensure, information from this systematic review was complemented with up-to-date information from those closest to the ground and to ensure true realities were well understood, for the final policy briefs, information was supplemented information from interviews and focus group discussions with key stakeholders performed by the NSOAP team. The results of these interviews were in line with the information presented in this review.

## Conclusion

This study represents a comprehensive review of a national SOA system according to the LCoGS NSOAP framework. The methods and structure used in this paper can serve as a preliminary step for other countries aiming to develop a NSOAP. The breadth of the review highlights this as a cost-effective way to gather baseline information to inform NSOAP, to supplement costlier on-the-ground baseline studies which due to resource constraints are likely to be narrower in scope. The review directly informed the priority areas for the NSOAP in Tanzania alongside baseline assessments and diverse stakeholder meetings and workshops.

## Appendix

### Search Terms

#### Embase (1000 Results on 12/28/16)

(‘Tanzania’/exp OR (‘Tanzania’ OR ‘Tanzanian’):ab,ti)

AND

(‘surgery’/exp OR ‘obstetric procedure’/exp OR ‘anesthesiological procedure’/exp OR ‘anesthesist’/exp OR ‘surgeon’/exp OR ‘obstetrician’/exp OR ‘nurse anaesthetist’/exp OR ‘nurse anaesthesia education’/exp OR ‘nurse midwife’/exp OR ‘nurse midwifery’/exp OR ‘nurse midwifery education’/exp OR ‘midwife’/exp OR ‘medical student’/exp OR ‘blood transfusion’/exp ‘blood safety’/exp OR ‘blood bank’/exp OR ‘operating room’/exp OR ‘operating room personnel’/exp OR ‘operating table’/exp OR ‘water supply’/exp OR ‘water management’/exp OR ‘physiotherapy’/exp OR ‘physical therapy education’/exp OR ‘quality control’/exp OR ‘biomedical engineering’/exp OR ‘medical technology’/exp OR ‘clinical laboratory’/exp OR ‘clinical laboratory personnel’/exp OR ‘instrument sterilization’/exp OR ‘anaesthesiology room general equipment’/exp OR ‘oxygen delivery device’/exp

OR

(‘surgical specialty’ OR ‘surgical specialties’ OR ‘surgical procedure’ OR ‘surgical procedures’ OR ‘anaesthesia’ OR ‘anaesthesia’ OR ‘anaesthesiology’ OR ‘anaesthesiology’ OR ‘anesthesiologist*’ OR ‘anaesthesiologist*’ OR ‘blood safety’ OR ‘transfusion’ OR ‘blood supply’ OR ‘operating room’ OR ‘operating rooms’ OR ‘operating theatre’ OR ‘operating theatres’ OR (‘electricity’ AND (‘surgery’ OR ‘surgeries’ OR ‘surgical’ OR ‘health’ OR ‘medical’ OR ‘medicine’):ab,ti):ab,ti OR ‘water supply’ OR ‘water purification’ OR ‘water recovery’ OR (‘water’ AND (‘surgery’ OR ‘surgeries’ OR ‘surgical’ OR ‘health’ OR ‘medical’ OR ‘medicine’):ab,ti):ab,ti OR ‘medical officer*’ OR ‘assistant medical officer*’ OR ‘clinical officer*’ OR ‘anaesthetist’ OR ‘anaesthetist’ OR ‘anaesthetists’ OR ‘anaesthetists’ OR ‘midwife’ OR ‘midwives’ OR ‘physiotherapy’ OR ‘physical therapy’ OR ‘quality improvement’ OR ‘quality control’ OR ‘biomedical engineering’ OR ‘biomedical technology’ OR ‘BMET’ OR ‘medical laboratory personnel’ OR ‘medical laboratory science’ OR ‘clinical laboratory’ OR ‘medical oxygen’ OR ‘oxygen supply’ OR ‘sterilization’ OR (‘sterilization’ AND (‘surgery’ OR ‘surgeries’ OR ‘surgical’ OR ‘health’ OR ‘medical’ OR ‘medicine’):ab,ti):ab,ti):ab,ti)

Added: blood bank, assistant medical officer, clinical officer, oxygen topics, medical student

#### Pubmed (1086 Results on 12/28/16)

(“Tanzania”[Mesh] OR “Tanzania”[tiab] OR “Tanzanian”[tiab]) AND (“Specialties, surgical”[Mesh] OR “surgical specialty”[tiab] OR “surgical specialties”[tiab] OR “Surgical Procedures, Operative”[Mesh] OR “surgical procedure”[tiab] OR “surgical procedures”[tiab] OR “Anaesthesia”[Mesh] OR “anaesthesia”[tiab] OR “anaesthesia”[tiab] OR “Anaesthesiology”[Mesh] OR “anaesthesiology”[tiab] OR “anaesthesiology”[tiab] OR “Anesthesiologists”[Mesh] OR “anesthesiologist”[tiab] OR “anaesthesiologist”[tiab] OR “Obstetric surgical procedures”[Mesh] OR “Obstetric nursing”[Mesh] OR “obstetric nursing”[tiab] OR “obstetric nurses”[tiab] OR “Blood safety”[Mesh] OR “blood safety”[tiab] OR “Blood transfusion”[Mesh] OR “transfusion”[tiab] OR “blood supply”[tiab] OR “Operating Rooms”[Mesh] OR “Operating Room Technicians”[Mesh] OR “Operating Room Nursing”[Mesh] OR “Operating Tables”[Mesh] OR “operating room”[tiab] OR “operating rooms”[tiab] OR “operating theatre”[tiab] OR “operating theatres” OR “Electricity/supply and distribution”[Mesh] OR (“electricity”[tiab] AND (“surgery”[tiab] OR “surgeries”[tiab] OR “surgical”[tiab] OR “health”[tiab] OR “medical”[tiab] OR “medicine”[tiab])) OR “Water supply”[Mesh] OR “water supply”[tiab] OR “Water purification”[Mesh] OR “water purification”[tiab] OR “water recovery”[tiab] OR (“water”[tiab] AND (“surgery”[tiab] OR “surgeries”[tiab] OR “surgical”[tiab] OR “health”[tiab] OR “medical”[tiab] OR “medicine”[tiab])) OR “medical officer”[tiab] OR “Nurse anaesthetists”[Mesh] OR “anaesthetist”[tiab] OR “anaesthetist”[tiab] OR “anaesthetists”[tiab] OR “anaesthetists”[tiab] OR “Nurse midwives”[Mesh] OR “midwife”[tiab] OR “midwives”[tiab] OR “Physical Therapy Specialty”[Mesh] OR “physiotherapy”[tiab] OR “physical therapy”[tiab] OR “Physical Therapy Modalities”[Mesh] OR “Quality improvement”[Mesh] OR “quality improvement”[tiab] OR “Quality Control”[Mesh] “quality control”[tiab] OR “Biomedical engineering”[Mesh] OR “biomedical engineering”[tiab] OR “Biomedical technology”[Mesh] OR “biomedical technology”[tiab] OR “BMET”[tiab] OR “Medical Laboratory Personnel”[Mesh] OR “medical laboratory personnel”[tiab] OR “Medical Laboratory Science”[Mesh] OR “medical laboratory science”[tiab] OR “Clinical Laboratory Services”[Mesh] OR “Clinical Laboratory Techniques”[Mesh] OR “clinical laboratory”[tiab] OR Sterilization[Mesh] OR “sterilization”[tiab] OR (“sterilization”[tiab] AND (“surgery”[tiab] OR “surgeries”[tiab] OR “surgical”[tiab] OR “health”[tiab] OR “medical”[tiab] OR “medicine”[tiab])))

#### African Index Medicus (38 Results on 12/28/16)

tanzania or tanzanian

and

surgery or surgeon or surgeons anaesthesia or anaesthesia or anaesthesiology or anaesthesiology or anesthesiologist or anaesthesiologist or anaesthesiologists or anesthesiologists or blood or transfusion or operating or operate or operating rooms or electricity or electrical or water or officer or anaesthetist or anaesthetist or anaesthetists or anaesthetists or midwife or midwives or physiotherapy or physiotherapist or oxygen or sterilization.

## References

[1] World Health Organization (2015) WHA 68.15: strengthening emergency and essential surgical care and anaesthesia as a component of universal health coverage. http://apps.who.int/gb/ebwha/pdf_files/WHA68/A68_R15-en.pdf

[2] Meara JG, Leather AJM, Hagander L, Alkire BC, Alonso N, Ameh EA (2015). Global surgery 2030: evidence and solutions for achieving health, welfare, and economic development. The Lancet.

[3] Burssa D, Teshome A, Iverson K, Ahearn O, Ashengo T, Barash D (2017). Safe surgery for all: early lessons from implementing a national government-driven surgical plan in Ethiopia. World J Surg.

[4] WHO (2018) Situation analysis of the health sector. WHO. http://www.who.int/healthsystems/publications/nhpsp-handbook-ch3/en/. Accessed Feb 9 2018

[5] Lancet Commission on Global Surgery Implementation Tools (2018) Lancet Comm Glob Surg. http://www.lancetglobalsurgery.org/implementation-tools. Accessed 9 Feb 2018

[6] Shamseer L, Moher D, Clarke M, Ghersi D, Liberati A, Petticrew M (2015). Preferred reporting items for systematic review and meta-analysis protocols (PRISMA-P) 2015: elaboration and explanation. BMJ.

[7] Tanzania Ministry of Health, Community Development, Gender, Elderly & Children (2018). http://moh.go.tz/en/. Accessed 9 Feb 2018

[8] World Bank (2018) World development indicators data. https://data.worldbank.org/. Accessed 9 Feb 2018

[9] Pereira C, Mbaruku G, Bergström S, McCord C, Nzabuhakwa C (2011). Emergency obstetric surgery by non-physician clinicians in Tanzania. Int J Gynecol Obstet.

[10] Luboga S, Matovu A, Macfarlane SB, Galukande M, von Schreeb J, Ozgediz D (2010). Essential surgery at the district hospital: a retrospective descriptive analysis in three african countries. PLoS Med.

[11] Ministry of Health and social Welfare (2013). Tanzania service availability and readiness assessment (SARA) 2012.

[12] Simba DO, Mbembati NAA, Museru LM, Lema LEK (2008). Referral pattern of patients received at the national referral hospital: challenges in low income countries. East Afr J Public Health.

[13] Ministry of Health and social Welfare (2007). Primary health services development programme—MMAM 2007–2017.

[14] Baker T, Irestedt L, Ulisubisya M, Jörnvall H (2016) Establishing an anaesthesia and intensive care partnership and aiming for national impact in Tanzania. Glob Health 12:7. http://www.embase.com/search/results?subaction=viewrecord&from=export&id=L609059108 10.1186/s12992-016-0144-1 http://sfx.hul.harvard.edu/sfx_local?sid=EMBASE&issn=17448603&id=doi:10.1186%2Fs12992-016-0144-1&atitle=Establishing+an+Anaesthesia+and+Intensive+Care+partnership+and+aiming+for+national+impact+in+Tanzania&stitle=Globalization+Health&title=Globalization+and+Health&volume=12&issue=1&spage=&epage=&aulast=Ulisubisya&aufirst=Mpoki&auinit=M.&aufull=Ulisubisya+M.&coden=&isbn=&pages=-&date=2016&auinit1=M&auinitm= PMC4799533 26993790

[15] Edler AA, Gipp MS (2010). Teaching nonphysician anesthesia providers in Tanzania: a movement toward sustainable healthcare development. Int Anesthesiol Clin.

[16] Cohen H, Cherian M, Groth S, Noel L, Mwakyusa DH, Penoyar T, et al (2012) Emergency and surgery services of primary hospitals in the United Republic of Tanzania. BMJ Open 2:e000369. http://www.embase.com/search/results?subaction=viewrecord&from=export&id=L364379841 10.1136/bmjopen-2011-000369 http://sfx.hul.harvard.edu/sfx_local?sid=EMBASE&issn=20446055&id=doi:10.1136%2Fbmjopen-2011-000369&atitle=Emergency+and+surgery+services+of+primary+hospitals+in+the+United+Republic+of+Tanzania&stitle=BMJ+Open&title=BMJ+Open&volume=2&issue=1&spage=&epage=&aulast=Penoyar&aufirst=Tom&auinit=T.&aufull=Penoyar+T.&coden=&isbn=&pages=-&date=2012&auinit1=T&auinitm= PMC3274714 22307096

[17] Mkony CA, Dicker RA, Beard JH, Akoko L, Mwanga A, Oresanya LB (2014). Surgical task-shifting in a low-resource setting: outcomes after major surgery performed by nonphysician clinicians in Tanzania. World J Surg.

[18] Epiu I, Tindimwebwa JVB, Mijumbi C, Chokwe TM, Lugazia E, Ndarugirire F (2017). Challenges of anesthesia in low- and middle-income countries: a cross-sectional survey of access to safe obstetric anesthesia in East Africa. Anesth Analg.

[19] Stafford RE, Morrison CA, Mahalu W, Godfrey G (2014). Challenges to the provision of emergency services and critical care in resource-constrained settings. Glob Heart.

[20] SIKIKA, The Medical Association of Tanzania (2013) Where are the doctors?—Tracking study of medical doctors

[21] Goodell AJ, Kahn JG, Ndeki SS, Kaale E, Kaaya EE, Macfarlane SBJ (2016) Modeling solutions to Tanzania’s physician workforce challenge. Glob Health Action, 9:31597. https://www.ncbi.nlm.nih.gov/pmc/articles/PMC4926102/10.3402/gha.v9.31597 PMC4926102 27357075

[22] WHO (2018) What are the latest statistics on health workforce availability? Why does the latest HRH report (A Universal Truth—No health without a Workforce, 2013) use a different threshold (33.45/10′000) from the WHO 2006 report (22.8/10′000)? WHO. http://www.who.int/workforcealliance/media/qa/05/en/. Accessed 3 Jul 2018

[23] Penoyar T, Cohen H, Kibatala P, Magoda A, Saguti G, Noel L (2012). Emergency and surgery services of primary hospitals in the United Republic of Tanzania. BMJ Open.

[24] Ministry of Health, Community Development, Gender, Elderly and Children. HFR WEB PORTAL—HomeAdvancedSearch Facilities (2018). http://hfrportal.ehealth.go.tz/index.php?r=facilities/homeAdvancedSearch. Accessed 10 Feb 2018

[25] Mama ye! Tanzania’s blood services: Fact sheet 2017. Dar es Salaam, United Republic of Tanzania: Mama ye!;

[26] Chalya PL, Gilyoma JM, Mabula JB, Kihunrwa A, Mbunda F, Massinde AN (2016) Blood transfusion practice in surgery at bugando medical centre in northwestern tanzania. Tanzan J Health Res 18. http://www.embase.com/search/results?subaction=viewrecord&from=export&id=L607798980 10.4314/thrb.v18i1.2 http://sfx.hul.harvard.edu/sfx_local?sid=EMBASE&issn=18216404&id=doi:10.4314%2Fthrb.v18i1.2&atitle=Blood+transfusion+practice+in+surgery+at+bugando+medical+centre+in+northwestern+tanzania&stitle=Tanzan.+J.+Health+Res.&title=Tanzania+Journal+of+Health+Research&volume=18&issue=1&spage=&epage=&aulast=Chalya&aufirst=Phillipo+L.&auinit=P.L.&aufull=Chalya+P.L.&coden=&isbn=&pages=-&date=2016&auinit1=P&auinitm=L

[27] Joseph AB, Akoko LO (2015) Blood utilization in elective surgery in a tertiary hospital in dar es salaam, Tanzania. Tanzan J Health Res 17. http://www.embase.com/search/results?subaction=viewrecord&from=export&id=L606824044 10.4314/thrb.v17i4.5 http://sfx.hul.harvard.edu/sfx_local?sid=EMBASE&issn=18216404&id=doi:10.4314%2Fthrb.v17i4.5&atitle=Blood+utilization+in+elective+surgery+in+a+tertiary+hospital+in+dar+es+salaam%2C+Tanzania&stitle=Tanzan.+J.+Health+Res.&title=Tanzania+Journal+of+Health+Research&volume=17&issue=4&spage=&epage=&aulast=Akoko&aufirst=Larry+O.&auinit=L.O.&aufull=Akoko+L.O.&coden=&isbn=&pages=-&date=2015&auinit1=L&auinitm=O

[28] Hollis AC, Ebbs SR (2016) An examination of inpatient medical record keeping in the Orthopaedic Department of Kilimanjaro Christian Medical Centre (KCMC), Moshi, Tanzania. Pan Afr Med J 23:207. http://www.embase.com/search/results?subaction=viewrecord&from=export&id=L610866388 10.11604/pamj.2016.23.207.8083 http://sfx.hul.harvard.edu/sfx_local?sid=EMBASE&issn=19378688&id=doi:10.11604%2Fpamj.2016.23.207.8083&atitle=An+examination+of+inpatient+medical+record+keeping+in+the+Orthopaedic+Department+of+Kilimanjaro+Christian+Medical+Centre+%28KCMC%29%2C+Moshi%2C+Tanzania&stitle=Pan+Afr.+Med.+J.&title=Pan+African+Medical+Journal&volume=23&issue=&spage=&epage=&aulast=Hollis&aufirst=Alexander+Conor&auinit=A.C.&aufull=Hollis+A.C.&coden=&isbn=&pages=-&date=2016&auinit1=A&auinitm=C PMC4907744 27347296

[29] Abuja Declaration (2017). http://www.un.org/en/africarenewal/vol15no1/151aids5.htm. Accessed 19 Dec 2017

[30] Luboga S, Hsia RY, Matovu A, Macfarlane SB, Galukande M, von Schreeb J (2010). Human resource and funding constraints for essential surgery in district hospitals in Africa: a retrospective cross-sectional survey. PLoS Med.

[31] WHO (2017) Strategic planning: transforming priorities into plans. http://www.who.int/healthsystems/publications/nhpsp-handbook-ch5/en/. Accessed 13 Dec 2017

[32] Coburger J, Leng LZ, Rubin DG, Mayaya G, Medel R, Ngayomela I (2014). Multi-institutional neurosurgical training initiative at a tertiary referral center in Mwanza, Tanzania: where we are after 2 years. World Neurosurg.

[33] Larsson E, Eriksson J, Baker T (2014). Quality of anaesthesia for caesarean sections at Muhimbili National Hospital, Dar es Salaam, Tanzania. Eur J Anaesthesiol.

[34] Clarke A, Blundell N, Forde I, Musila N, Spitzer D, Naqvi S (2010). Can guidelines improve referral to elective surgical specialties for adults? A systematic review. Qual Saf Health Care.

[35] Tiska MA, Adu-Ampofo M, Boakye G, Tuuli L, Mock CN (2004). A model of prehospital trauma training for lay persons devised in Africa. Emerg Med J EMJ.

[36] Jayaraman S, Mabweijano JR, Lipnick MS, Caldwell N, Miyamoto J, Wangoda R (2009). First things first: effectiveness and scalability of a basic prehospital trauma care program for lay first-responders in Kampala. Uganda PLoS One.

[37] Hsia RY, Mbembati NA, Macfarlane S, Kruk ME (2012). Access to emergency and surgical care in sub-Saharan Africa: the infrastructure gap. Health Policy Plan.

[38] World Health Organization (WHO) (2018) Increasing access to health workers in remote and rural areas through improved retention. WHO. http://www.who.int/hrh/retention/guidelines/en/. Accessed 10 Feb 2018 23741785

[39] Ashengo T, Skeels A, Hurwitz EJH, Thuo E, Sanghvi H (2017) Bridging the human resource gap in surgical and anesthesia care in low-resource countries: a review of the task sharing literature. Hum Resour Health 15:77. https://www.ncbi.nlm.nih.gov/pmc/articles/PMC5688799/10.1186/s12960-017-0248-6 PMC5688799 29115962

[40] Ministry of Health and Social Welfare (MoHSW) (2013) Tanzania National eHealth Strategy 2013–2018. http://ihi.eprints.org/3727/. Accessed 10 Feb 2018

[41] Center for Global Health Delivery—Dubai, Harvard Medical School (2018) National surgical obstetric and anaesthesia planning: process and consensus recommendations. Program Glob Surg Soc Change. https://docs.wixstatic.com/ugd/d9a674_a8367aa7b82642ac81f67fb435660801.pdf. Accessed 24 Jun 2018

